# Editorial: Assistive technologies in aging and disability

**DOI:** 10.3389/fpubh.2026.1798147

**Published:** 2026-04-14

**Authors:** Jamie Danemayer, Maryam Bandukda, Hasheem Mannan, Suraj Singh Senjam

**Affiliations:** 1 Department of Computer Science, University College London, London, United Kingdom; 2 University College Dublin, Dublin, Ireland; 3 School of Nursing, Midwifery, and Health Systems, All India Institute of Medical Sciences, New Delhi, India

**Keywords:** aging, assistive technology, digital health, digital literacy, disability, equity, inclusive design, user-centered design

## Introduction

Population aging is driving the reorientation of health and social care systems globally (1), intensifying the demand for assistive technologies (AT) and access to digital health tools that can support independence, participation, and wellbeing (2, 3). While this Research Topic focuses primarily on older adults, the contributions represent a broader spectrum: access to assistive technology throughout the life course is part of equitable access to healthy and active lifestyles, and dignified aging for all (McVeigh; Borg et al.; Alfalih and Ragmoun). Without consideration for systemic inequities in accessing AT, emerging evidence and innovations related to AT risk amplifying these disparities rather than alleviating them (McVeigh; Borg et al.; Shao et al.; Kennedy et al.; Zhang et al.; Meraya et al.; Ko et al.; Requena et al.). This special edition brings together a diverse collection of articles reflecting a wide range of populations for whom aging and AT are urgent priorities. Common topics of representation and equity, digital literacy, smart systems, and privacy, explored within data collection and research design that are both user-centered and psychosocially-informed, unify these 15 studies.

## Representation and equity

A central need in disability-inclusive aging research is the capacity to distinguish between the unique experiences of aging with a disability, compared to acquiring disability as a result of the aging process. Improving the representation of both experiences in data and evidence enables more appropriate policy and innovation advancements in AT that address different support needs and structural barriers. Three contributions indicate that failure to attend to these differences can lead to inequitable outcomes in social participation, healthcare access, and the widening of digital exclusion (McVeigh; Shao et al.; Ko et al.). As global population longevity accelerates aging, addressing these socio-demographic differences in research settings is critical to avoiding exclusionary practices downstream, such as the generation of inappropriate health guidance or unusable AT (McVeigh; Kennedy et al.).

Digital health literacy and the widening digital divide appear across this topic as critical determinants of whether innovations translate into improved outcomes (Shao et al.; Meraya et al.; Ko et al.; Wang, Xiang et al.). Studies consistently report low levels of digital health literacy among older adults, particularly those with chronic health conditions or disabilities, shaped by personal, economic, social, and environmental factors (Shao et al.; Zhang et al.), such as inequitable access to broadband and digital devices (Shao et al.). Without deliberate efforts to improve accessibility, usability, and support, digital health risks reinforce a type of “data poverty,” wherein older adults and people with disabilities remain underrepresented in research and emerging data and become locked out of benefitting from subsequent advances in policies and technical innovation (Borg et al.; Zhang et al.). These concerns are powerfully illustrated by a case study on inaccessible COVID-19 home tests, which revealed substantial gaps in resources for accessible product development during a public health emergency (Kennedy et al.). While the initiative successfully improved test accessibility, it also generated expert networks, documented best practices, and validated a framework for future accessibility-focused innovation, demonstrating how equity-oriented approaches can yield lasting benefits beyond crisis response (Kennedy et al.).

Equity considerations extend beyond health and AT into legal, social, and occupational domains. One contribution examines the role of AT in fulfilling disability justice as a fundamental human right, outlining its importance at the population- and individual-level for operationalising Article 13 of the UN Convention on the Rights of Persons with Disabilities (McVeigh). A multi-country study further analyzed how gender and national development context intersect to shape access to assistive products, highlighting the need for intersectional data collection and equity-driven approaches to policymaking (Borg et al.).

## Smart systems and privacy

Rapid advances in digital, Artificial Intelligence (AI)-enabled, and sensor-based technologies introduce opportunities as well as risks. Smart systems promise scalable solutions to workforce shortages in long-term care by alleviating some caregiving tasks (McVeigh; Xue; Mazuz and Yamazaki). However, their success depends on aligning with real-world needs and earning trust from users. Across this topic, authors emphasize that technical innovation must not outpace users' willingness or ability to engage, and that specific design considerations are needed for populations with low digital literacy or limited access to technology (Shao et al.; Zhang et al.; Meraya et al.; Ko et al.). In this sector, ambient data collection and continuous monitoring systems are emerging as useful tools for primary users, caregivers, and researchers, yet privacy concerns with such systems remain a key barrier to scaling up. For example, research applying the World Health Organization's Intrinsic Capacity framework shows how wearable motion sensors can capture locomotion, vitality, cognition, and psychological and sensory dimensions of functional ability, offering potential for early detection, anomaly identification, and personalized intervention (Ju et al.). Similarly, reviews of sensor-based applications to enhance independence in daily life illustrate a progression from fall detection to predictive modeling and preventative care, as well as personalized interventions that are increasingly capable of meaningfully engaging with the social and emotional dimensions of aging, in addition to the physical and cognitive dimensions (Requena et al.). While predictive and personalized capabilities are valuable for proactive planning and reducing reliance on intensive medical services, authors also caution that the use of AI in monitoring raises ethical and privacy concerns among users that must be carefully addressed, especially in shared or multi-resident environments (Requena et al.). These approaches also raise critical questions about how data are collected, governed, and protected, particularly among vulnerable older adults with limited digital literacy and capacity to provide informed consent (Alfalih and Ragmoun; Ju et al.). Broader analyses of smart systems for the care of older adults, including a review of developments in China, similarly highlight applications in monitoring, smart homes, emotional companionship, and medical services, while emphasizing the need to simplify interfaces and strengthen data privacy to ensure sustainable implementation (Xue).

## User-centered and psychosocially-informed design

User-centered design processes that foreground a meaningful understanding of user needs are foundational to this topic. They exemplify a method of building trust and aligning priorities between designers and users. One contribution applies the double-diamond model (discover, define, develop, and deliver) to the design of mobility scooters for older adults, demonstrating how structured innovation frameworks can improve design efficiency while remaining responsive to dynamic needs, such as those related to aging societies (Wang et al.). Research in telehealth also touches on the importance of trust and the role of co-design, where users are active participants in the design process. Research from Saudi Arabia found that fear or dislike of technology was a major barrier to telehealth use among older adults, highlighting the need for user-friendly designs and trust-building to increase adoption (Meraya et al.). One contribution from the United Kingdom similarly reports mixed experiences among people with disabilities, with benefits including improved accessibility, but persistent barriers related to connectivity, device access, and some disability-specific challenges, particularly for users with sensory impairments (Ko et al.). These studies agree that telehealth should complement, rather than replace, face-to-face care through hybrid models and should be co-designed with users with unique needs (Meraya et al.; Ko et al.).

Emerging AT are frequently piloted in the context of physical functioning and rehabilitation, with research illustrating the complexity of translating new innovations into meaningful outcomes. Engaging with psychosocial dimensions of wellbeing, as emphasized across this topic (Alfalih and Ragmoun; Shao et al.; Zhang et al.; Meraya et al.; Ko et al.; Requena et al.; Xue; Ju et al.; Cai et al.), is a critical component to design efficacy that has historically been underrepresented in design research. One contribution examining virtual reality-based rehabilitation for stroke survivors shows that adoption is shaped as much by emotional, motivational, and social factors as by technical performance (Cai et al.). A qualitative study examining companion robots in dementia care demonstrates how technology can relieve some caregiving tasks, provided caregivers are empowered to use it effectively (Mazuz and Yamazaki). Instead of replacing human care, robot-assisted sessions fostered stimulation, engagement, reciprocity, and shared emotional experiences, supporting a person-centered approach for people living with mild cognitive impairment and dementia (Mazuz and Yamazaki). Another contribution demonstrates how transformational leadership strategies enable better integration of AT in workplaces and, as a result, foster self-confidence and self-efficacy among AT users, as measured by individual wellbeing and a sense of inclusion rather than a sole focus on productivity (Alfalih and Ragmoun). These findings characterize AT not as standalone tools, but as interventions embedded within broader social and care ecosystems, and further emphasize the utility of a holistic understanding of AT: one that integrates emotional and psychological wellbeing, social context, individual preferences, and structural constraints as core determinants of adoption and impact (Shao et al.; Wang, Xiang et al.; Mazuz and Yamazaki; Cai et al.).

## Conclusion

This editorial concludes the Research Topic by emphasizing that innovation in assistive technology for aging and disability must advance within a framework that upholds representation, equity, privacy and inclusive design practices. Factoring emotional and social dimensions into AT research and development is critical to effectively expanding AT access and adoption. The contributions presented here offer not only technological and population insights but also guidance on research design to better support equitable access to AT amid increasing global demand.
